# P2X7 Nucleotide and EGF Receptors Exert Dual Modulation of the Dual-Specificity Phosphatase 6 (MKP-3) in Granule Neurons and Astrocytes, Contributing to Negative Feedback on ERK Signaling

**DOI:** 10.3389/fnmol.2017.00448

**Published:** 2018-01-10

**Authors:** Mª José Queipo, Juan C. Gil-Redondo, Verónica Morente, Felipe Ortega, Mª Teresa Miras-Portugal, Esmerilda G. Delicado, Raquel Pérez-Sen

**Affiliations:** Departamento de Bioquímica y Biología Molecular IV, Facultad de Veterinaria, Instituto Universitario de Investigación en Neuroquímica, Instituto de Investigación Sanitaria del Hospital Clínico San Carlos, Universidad Complutense Madrid, Madrid, Spain

**Keywords:** astrocytes, DUSP6, ERKs, glial cells, granule neurons, MKP3, P2X7 receptors

## Abstract

Extracellular signal-regulated kinases 1 and 2 (ERK1/2) play a central role in the intracellular signaling of P2X7 nucleotide receptors in neurons and glial cells. Fine spatio-temporal tuning of mitogen-activated protein (MAP) kinases is essential to regulate their biological activity. MAP kinase phosphatases (MKPs) are dual specificity protein phosphatases (DUSPs) that dephosphorylate phosphothreonine and phosphotyrosine residues in MAP kinases. This study focuses on how DUSP, DUSP6/MKP3, a phosphatase specific for ERK1/2 is regulated by the P2X7 nucleotide receptor in cerebellar granule neurons and astrocytes. Stimulation with the specific P2X7 agonist, BzATP, or epidermal growth factor (EGF) (positive control for ERK activation) regulates the levels of DUSP6 in a time dependent manner. Both agonists promote a decline in DUSP6 protein, reaching minimal levels after 30 min yet recovering to basal levels after 1 h. The initial loss of protein occurs through proteasomal degradation, as confirmed in experiments with the proteasome inhibitor, MG-132. Studies carried out with Actinomycin D demonstrated that the enhanced transcription of the *Dusp6* gene is responsible for recovering the DUSP6 protein levels. Interestingly, ERK1/2 proteins are involved in the biphasic regulation of the protein phosphatase, being required for both the degradation and the recovery phase. We show that direct Ser^197^ phosphorylation of DUSP6 by ERK1/2 proteins could be part of the mechanism regulating their cytosolic levels, at least in glial cells. Thus, the ERK1/2 activated by P2X7 receptors exerts positive feedback on these kinase’s own activity, promoting the degradation of one of their major inactivators in the cytosolic compartment, DUSP6, both in granule neurons and astrocytes. This feedback loop seems to function as a common universal mechanism to regulate ERK signaling in neural and non-neural cells.

## Introduction

The mitogen-activated protein (MAP) kinases extracellular signal-regulated kinases 1 and 2 (ERK1/2) regulate multiple processes in neural cells, such as the balance between survival and cell death, proliferation and differentiation, and they also play a crucial role during nervous system development and in neuroregeneration. Numerous factors modulate ERK1/2 signaling, alone or in combination, including extracellular signals, scaffolding and signaling proteins recruited to specific cascades, and other intrinsic tissue or cell factors. Moreover, the spatio-temporal regulation of the ERK1/2 pathway shapes the final biological response to its activation. In neural models, transient or sustained early activation of ERK1/2 signaling is involved in either proliferation or differentiation, respectively. By contrast, prolonged and delayed ERK1/2 activation is more closely related to cell death following certain toxic stimuli (Marshall, 1995; Chu et al., 2004). Hence, fine tuning of ERK1/2 signaling is necessary to obtain adequate responses.

Different upstream and downstream mechanisms influence the activation kinetics of ERK1/2 signaling (Junttila et al., 2008). Among those involved in their negative regulation, MAPK protein phosphatases (MKPs) are considered crucial elements that determine the final duration and magnitude of MAPK signaling (Dickinson and Keyse, 2006; Patterson et al., 2009). There are 10 dual specificity protein phosphatases (DUSPs) in the MKP family that specifically dephosphorylate Ser/Thr and Tyr residues within the activation loop of MAPKs. DUSPs can be classified according to their substrate selectivity (ERK1/2, p38 and JNK MAPKs) and their subcellular localization. Understandably, the regulation of these phosphatases is complex, particularly since the same MAPKs that represent their substrates can also regulate their expression, implicating them in a negative feedback loop that offers some control over MAPK signaling (Dickinson and Keyse, 2006; Patterson et al., 2009).

DUSP expression and activity are tightly regulated at different levels, and finally depend on cell type and specific cellular context. Regulatory mechanisms involve several processes at the transcriptional level, but also cover post-transcriptional and post-translational events. Actually, DUSP expression is rapidly increased upon growth factor receptor stimulation. Several transcription factors activated by one or more MAPK pathways are responsible for the rapid expression of *Dusp* genes. This implies that MAPKs acting as DUSP substrates are responsible for the transcriptional induction of *Dusp* genes, functioning in negative feedback loops, and in crosstalk between distinct MAPK modules (Ekerot et al., 2008; Zeliadt et al., 2008). In addition, for some inducible DUSPS, stress conditions/factors that induce stress can also favor and activate its transcriptional expression (Staples et al., 2010; Bermudez et al., 2011). At the second level, post-transcriptional regulation, involve the participation of several RNA binding proteins that have the ability to stabilize *Dusp* mRNA increasing its half-life, which also accounts for increases in the DUSP6 protein levels (Kuwano et al., 2008; Bermudez et al., 2011). On the other hand, specific micro-RNAs can also promote *Dusp* gene silencing (Zhu et al., 2010). In some aspects, MAPK themselves also have the ability to mediate both DUSP transcription and mRNA stabilization through its 3′-UTR (Bermudez et al., 2011). The third mode of control takes place by post-translational modifications that rapidly affects DUSP turnover, thereby providing a dynamic control over MAPK signaling. DUSP1 and DUSP6 provide numerous examples of phosphorylation processes in different key residues that account for either protein stabilization or increase in the rate of protein degradation. Not only MAPKs, but also different types of signaling kinases participate in this regulatory process, such as mTOR, Protein kinase C (PKC) and CK2 (Marchetti et al., 2005; Choi et al., 2006; Lin and Yang, 2006; Bermudez et al., 2008; Cagnol and Chambard, 2010).

One of the most representative members of the DUSP family that selectively dephosphorylates ERK1/2 is DUSP6, also termed MKP-3 (Muda et al., 1996; Camps et al., 1998). This is a constitutive protein phosphatase that regulates the basal levels of phosphorylated ERKs in the cytosolic compartment, and that is a negative regulator of mitogen signaling (Ekerot et al., 2008). Indeed, although it does not behave as an immediate early gene, *Dusp6* can be induced transcriptionally by the Ras/MEK/ERK cascade triggered by growth factors like FGF, albeit with delayed kinetics. ERK activation also regulates DUSP6 protein expression and stability through mechanisms that involve direct phosphorylation and protein complex assembly (Marchetti et al., 2005). However, it should be noted that most studies into DUSP6 regulation have been carried out in exogenous overexpression systems, cell lines overexpressing the phosphatase or tumor cells. Regarding the latter, it remains unclear how this protein phosphatase may be implicated in cancer. While DUSP6 expression is enhanced in some kinds of cancer (melanoma and glioma cells), suggesting it may be a tumor promotor (Messina et al., 2011; Li et al., 2012), its expression is dampened in other tumors where it may exert a role as a tumor suppressor, such as pancreatic invasive cancer, primary lung cancer and ovarian cancer (Furukawa et al., 2003; Chan et al., 2008; Okudela et al., 2009). Significantly, very little is known about the intrinsic regulation of DUSP6 in native tissues and its modulation by endogenous extracellular signals that activate receptor tyrosine kinases (RTKs) e.g., mitogens (Ham et al., 2015; Donaubauer et al., 2016).

In an attempt to investigate the role of endogenous DUSP6 in nervous system, we took advantage of earlier studies carried out on primary cultures of granule neurons and astrocytes obtained from the rat cerebellum. ERK1/2 signaling cascades are well characterized in both these cell types and they are activated by stimulating different kinds of growth factor receptors or G protein-coupled receptors (GPCRs) (Delicado et al., 2005; Subramaniam et al., 2005). Interestingly, an ionotropic receptor, the P2X7 nucleotide receptor that is activated by high extracellular concentrations of ATP, is attracting growing interest in both these cell models. Indeed, ERK1/2 activation elicited by stimulation of the P2X7 receptor underlies the neuroprotection against glutamate excitotoxicity driven by this receptor (Ortega et al., 2011). ERK activation by P2X7 receptors in granule neurons is dependent on the entry of extracellular calcium and calcium/calmodulin kinase II (CaMKII) activation. Conversely, ERK activation in cerebellar astrocytes was not dependent on extracellular calcium nor does it involve nPKC isoforms (Carrasquero et al., 2010; Ortega et al., 2011). Here, we investigated the role of endogenous DUSP6 phosphatase in regulating ERK1/2 signaling, both in basal conditions and when P2X7 receptors are stimulated. Moreover, the regulation of DUSP6 by epidermal growth factor (EGF) receptors was taken as a positive control of growth factor regulation of this phosphatase. This study set out to determine if DUSP6 is specifically involved in regulating cytosolic ERK1/2 signaling in these two cellular types, and whether it is regulated by nucleotide receptors in a similar fashion as it is by growth factors.

## Materials and Methods

### Chemicals, Materials and Antibodies

Papain was purchased from Worthington (Lake Wood, NJ, USA), while Neurobasal medium, the B-27 supplement, fetal calf serum (FCS) and other culture reagents were obtained from GIBCO (Life Technologies, Barcelona, Spain). Plastic Petri dishes and culture flasks were supplied by Falcon Becton Dickinson Labware (Franklin Lakes, NJ, USA). Actinomycin D, antibiotics, Cytosine β-D-arabinofuranoside (AraC), Dulbecco’s Modified Eagle Medium (DMEM), nucleotides, EGF, (E)-2-benzylidene-3-(cyclohexylamino)-2,3-dihydro-1H-inden-1-one (BCI), and poly-D-lysine were all purchased from Sigma Aldrich (St. Louis, MO, USA). U0126 was obtained from Calbiochem Corporation (San Diego, CA, USA), and specific antibodies against phospho-ERK1/2 (Tyr^204^) and MKP3 were purchased from Santa Cruz Biotechnology (Santa Cruz, CA, USA) and Abcam (Cambridge, UK), respectively. The anti-MKP-3 (Phospho-Ser197) antibody was from Signalway Antibody (Washington, DC, USA). Antisera for Glyceraldehyde-3-phosphate dehydrogenase (GAPDH) was generated in rabbit and purchased from Cell Signaling Technology (Beverly, MA, USA). Anti-rabbit GFAP was from Dako (Denmark), anti-mouse GFAP, and anti-mouse anti-βIII tubulin were from Sigma. Secondary horseradish peroxidase-conjugated anti-mouse and anti-rabbit antibodies were from Dako, while the anti-rabbit and anti-goat equivalents were from Dako (Denmark: anti-rabbit, anti-goat) and Synaptic Systems (Goettingen, Germany: donkey anti-rabbit IgG Cy3-conjugated), respectively. Anti-mouse FITC and anti-rabbit Cy3 from Jackson ImmunoResearch (Suffolk, UK). All other reagents not specified were routinely supplied by Sigma, Merck (Darmstadt, Germany) or Roche Diagnostics SL (Barcelona, Spain).

### Experimental Animals

All experiments were carried out at the Complutense University of Madrid (Madrid, Spain) following the International Council for Laboratory Animal Science guidelines. All the procedures were approved by both the Animal Ethics Committee of Complutense University and the Regional Government of Madrid (Area of Animal Protection) according to RD 53/2013 (Spanish Government) related to the European Directive 2010/63/UE on the protection of animals used for scientific purposes. The assays were designed to minimize the number of animals used while maintaining statistical validity.

### Primary Cell Cultures

Primary cultures of cerebellar granule neurons and astrocytes were prepared as described previously (Morente et al., 2014). Briefly, the cerebellum was removed aseptically from Wistar rat pups (P7) and digested with papain. For neuronal cultures, the dissociated cells were resuspended in Neurobasal medium supplemented with B-27, 21 mM KCl, 2 mM glutamine, 100 U/ml penicillin, 0.1 mg/ml streptomycin and 0.25 μg/ml amphotericin B and plated at a density of 200,000 cells/cm^2^ in plastic Petri dishes or onto glass coverslips precoated with 0.1 mg/ml poly-D-lysine. The cells were maintained in a humidified incubator at 37°C in an atmosphere of 5% CO_2_ for 8–10 days, and AraC (10 μM) was added 24 h after plating to avoid the proliferation of glial cells. Under these conditions, granule cell culture purity was estimated to be higher than 95%.

For astrocyte cultures, the cerebellar cells were resuspended in DMEM containing 10% (v/v) FCS, 25 mM glucose, 2 mM glutamine, 100 U/ml penicillin, 100 mg/ml streptomycin and 2.5 μg/ml amphotericin, and they were plated in culture flasks at a density of 70,000 cells/cm^2^. The cells were maintained in culture until they reached confluence (approximately 10–12 days), replacing the medium every 3–4 days. Astrocyte cultures were depleted of microglial cells by orbital shaking and the astrocytes were then detached from the culture flasks by trypsin digestion and seeded onto culture plates. Absence of immunostaining for the microglia marker, Iba-1, was routinely assessed in astrocytic cultures to confirm the lack of contamination with microglial cells. For Western blotting and RT-QPCR studies, the astrocytes were plated onto Petri dishes at a density of 35,000 cells/cm^2^ and for immunocytochemistry experiments, they were plated onto coverslips in 35 mm Petri dishes at a density of 50,000 cells/cm^2^. Astrocytes were routinely used 48 h after plating.

### Cell Treatments and Lysate Preparation

Cerebellar granule neuron cultures were used at 8–10 DIV (days *in vitro*). Neurons were stimulated with the nucleotide or other agents by adding the corresponding compounds to the culture medium. In the case of astrocyte cultures, the culture medium was replaced with DMEM containing 1% FCS 24 h after plating and the cells were stimulated by adding the agents to this medium. In both cases the cells were maintained in the incubator for the times indicated and the incubations were stopped by removing the medium prior to lysing the cells in cold lysis buffer: 20 mM MOPS, 50 mM NaF, 40 mM β-glycerophosphate, 1 mM sodium orthovanadate, 5 mM EDTA, 2 mM EGTA, 0.5% Triton X-100 [pH 7.2], 1 mM PMSF and protease inhibitor cocktail.

### Western Blotting

Total cell lysates (15–30 μg protein) were resolved containing 0.1% Tween-20 by SDS-PAGE and transferred to PVDF membranes. The membranes were blocked in low-fat milk powder (5%) in TBS, and then incubated overnight at 4°C with primary antibodies diluted either in TBS containing 0.1% Tween-20 or in bovine serum albumin (3% BSA w/v) dissolved in TBS containing 0.1% Tween-20. The primary antibodies were used at the following dilutions: anti-phospho-ERK1/2, anti-total ERK (1:1000), anti-MKP3 (1:1000), anti-phospho-MKP3 (1:1000), anti-GAPDH (1:10,000). Antibody binding was detected for 1 h at room temperature with anti-mouse (1:2000) and anti-rabbit (1:1000) horseradish peroxidase-conjugated secondary antibodies, and visualized by the ECL method (Kit Western Lighting ECL PRO, Perkin Elmer, Madrid, Spain). Chemiluminescence images were obtained with the ImageQuant LAS 500^®^ image system and quantified by densitometry using the ImageQuantTL software.

### Quantitative Real Time-PCR (Q-PCR)

Total RNA was extracted from the cultured cells using the Speedtools Total RNA extraction kit (Biotools) and 1 μg DNAse-treated RNA (Turbo-DNA free, Ambion) was then used for first strand cDNA synthesis, according to the manufacturer’s instructions (TaqMan Reverse Transcript Reagents, Applied Biosystems kit). Quantitative real time PCR (Q-PCR) was performed using Applied Biosystems Step One Plus and with specific forward and reverse primer pairs from Roche Applied Science (Universal ProbeLibrary Probes labeled with FAM): TCT CTGATCACTGGAGCCAAA/GTTTTTGCCTCGGGCTTC for *Dusp6*; and CCCCTCTGGAAAGCTGTG/GGATGCAGGGAT GTTCT for *Gapdh*. Amplification was performed using a LuminoCt^®^ Qpcr Ready Mix™ (Sigma), and employing 40 cycles of denaturalization at 95°C for 20 s, synthesis at 60°C for 20 s. Quantifications were normalized to *Gapdh* expression and the results are expressed relative to the control conditions in the absence of any stimulation.

### Immunocytochemistry

Cells plated on coverslips were stimulated as described previously, washed with PBS and fixed in 4% paraformaldehyde for 15 min at room temperature. After washing three times with PBS, the cells were permeabilized with 0.2% Triton X-100 and blocked with 2% BSA in PBS for 1 h at room temperature. The cells were then incubated overnight at 4°C with primary antibodies against either MKP3 or pERK (1:100), anti-βIII tubulin (1:1000) and anti-GFAP (1:500). Subsequently, the cells were washed with PBS and incubated with FITC (1:200) or Cy3 (1:400) conjugated secondary antibodies for 1 h at room temperature. The nuclei were counterstained with DAPI (Invitrogen, Barcelona, Spain) and the coverslips were mounted onto glass slides using Aqua-Poly/Mount (Polysciences Europe GmbH, Bergstrasse, Germany). Confocal images were acquired on a TCS SPE microscope from Leica Microsystems (Wetzlar, Germany) and they were analyzed using the ImageJ and Leica LAS AF Lite software.

### Statistical Analysis

The results are expressed as the means ± SEM calculated from at least three experiments performed on cells from different cultures. Statistical differences between two groups of data were assessed using a *t*-test and a *p* value < 0.05 was taken as the limit of significance. When multiple comparisons were made, one-way analysis of variance was used and a Dunnett’s post-test analysis was applied only when a significant (*p* < 0.05) effect was evident (GraphPad Prism 5; GraphPad Software Inc., San Diego, CA, USA).

## Results

### P2X7 and EGF Receptor Stimulation Regulates the Expression of DUSP6 in Cerebellar Granule Neurons and Astrocytes

**Figure 1:**
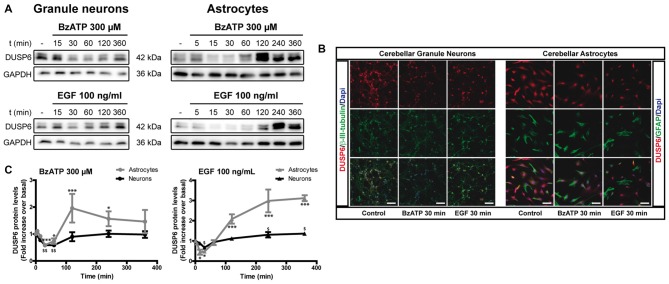
BzATP and epidermal growth factor (EGF) regulate the levels of dual specificity protein phosphatase 6 (DUSP6) protein in a time-dependent manner. Cerebellar granule neurons or astrocytes were exposed to BzATP (300 μM: •, 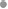) or EGF (100 ng/mL: ▲, 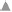) for different times and DUSP6 and Glyceraldehyde-3-phosphate dehydrogenase (GAPDH) were detected in the total lysates by immunoblotting. **(A)** Immunoblots of a representative time-course experiment for each effector. **(B)** Cells were incubated with or without the effectors, 300 μM BzATP or 100 ng/mL EGF, for 30 min, fixed and the presence of DUSP6 was detected by immunocytochemistry. Representative immunofluorescence images are shown. Scale bars represent 50 μm. **(C)** The diagrams represent the quantification of the time courses obtained by normalization to the corresponding GAPDH levels. The data are represented as the means ± SEM from three independent experiments performed on different cultures. ***p < 0.001; ^$$^*p* < 0.01; ^$^,*p < 0.05.

**Figure 2:**
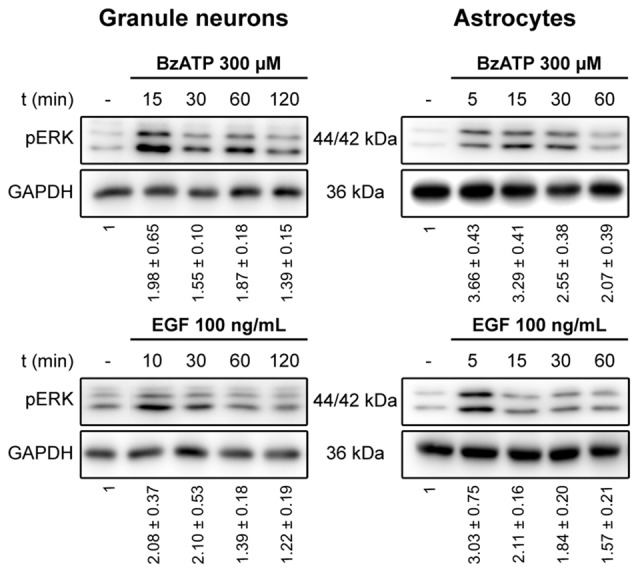
Time course for ERK activation stimulated by BzATP and EGF in cerebellar granule neurons and astrocytes. Cells were stimulated with BzATP (300 μM) or EGF (100 ng/mL) for the times indicated and the phosphorylated extracellular signal-regulated kinases 1 and 2 (ERK1/2) was detected by immunoblotting. The blots shown are representative of the independent experiments performed on different cultures. The values represent the quantification of pERK after normalization to the corresponding GAPDH levels and they are the means ± SEM from the data obtained from the independent experiments (*n* = 7).

**Figure 3:**
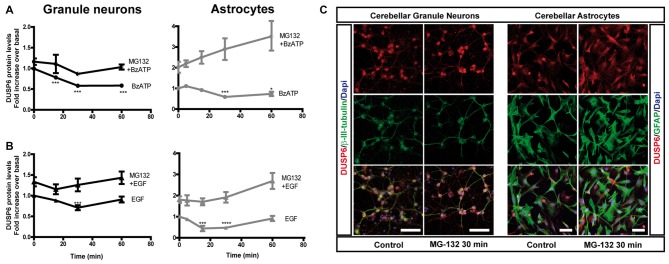
Effect of inhibiting the proteasome on the changes in DUSP6 protein induced by BzATP and EGF. Cells were maintained in the presence or absence of MG-132 (10 μM) for 30 min prior to stimulation with BzATP (300 μM) **(A)** or EGF (100 ng/mL) **(B)** for the times indicated, and immunoblots were probed for DUSP6 for granule neurons and astrocytes. The diagrams represent the quantification of the time courses obtained by normalization to the corresponding GAPDH levels. The data represent the means ± SEM from four independent experiments performed on different cultures.** (C)** Cells plated on coverslips were treated with or without MG-132 (10 μM, 30 min), fixed and the presence of DUSP6 was detected by immunocytochemistry (see “Materials and Methods” section). Representative immufluorescence images of granule neurons and astrocytes were shown. Scale bars represent 50 μm. ****p < 0.0001; ***p < 0.001; *p < 0.05.

We previously demonstrated that stimulation of the P2X7 receptor in cerebellar granule neurons and astrocytes was associated with ERK activation quantified in western blots probed with phospho-specific antibodies (Jiménez et al., 2002; Ortega et al., 2011). In both cases, maximal activation was achieved after a 10–15 min stimulation and it decreased after more prolonged stimulation with the specific P2X7 agonist, BzATP. We assessed whether the increase in ERK phosphorylation was accompanied by changes in DUSP6 (MPK-3) expression in western blots. Since the main inducers of MKP expression are their own substrates, MAP Kinases, we used EGF as a positive control as it produces considerable ERK activation in cerebellar astrocytes (Jiménez et al., 2002). Stimulation of either cell type with BzATP (300 μM) or EGF (100 ng/mL) regulated the DUSP6 protein levels in a biphasic pattern (Figure 1). P2X7 and EGF receptor stimulation promoted a rapid decline in the DUSP6 protein, starting as soon as 15 min with decreases around 20%–25%, and reaching a minimum decline of around 45% after 30 min (first phase; see also Figures 3, 10). These changes in DUSP6 protein levels were also detected by immunocytochemistry (Figure 1B). Subsequently, there was an accumulation of DUSP6, which recovered towards basal levels after longer incubations, and even increased 3-fold over the controls in cerebellar astrocytes (second phase). The estimated half-life of DUSP6 (first phase) following nucleotide stimulation was around 17.5 min, and it was similar in neurons and astrocytes. By contrast, the kinetics of recovery of this phosphatase (second phase) differed and it was quite rapid in the case of EGF in glial cells as the basal protein levels recovered after 1 h in the presence of this growth factor. However, the recovery of basal levels of DUSP6 was slower when stimulated with BzATP, requiring almost 2 h in the presence of the agonist. This phosphatase recovery phase may correspond to the synthesis of new protein. Moreover, an inverse relationship between the DUSP6 protein and ERK phosphorylation triggered by the two agonists was evident over time in both cell types (Figure 2). It appears that decline in pERK activity is preceding DUSP6 recovery, especially at stimulation periods of 30 min with BzATP. Therefore it cannot be excluded that other protein phosphatases may be involved in deactivation of ERK1/2 signaling.

**Figure 4:**
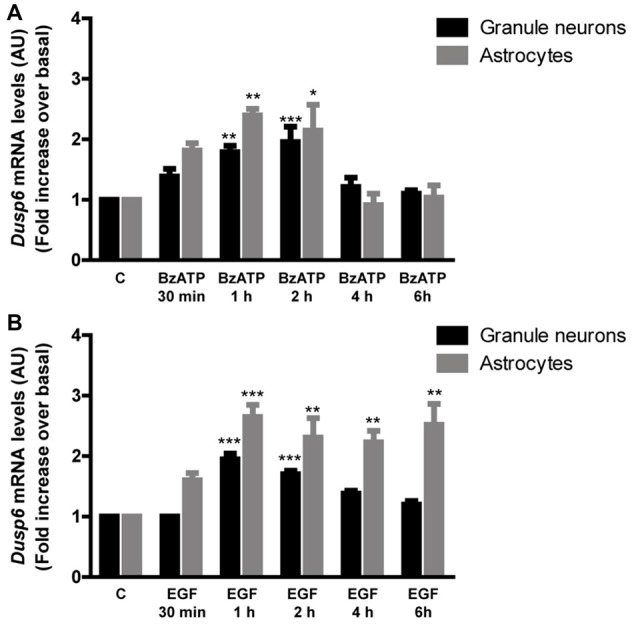
BzATP and EGF increase *Dusp6* mRNA levels in cerebellar granule neurons and astrocytes. Cells were stimulated with the nucleotide (300 μM) **(A)** or the growth factor (100 ng/mL) **(B)** for the times indicated. The total RNA isolated from the cells was reverse transcribed and the presence of *Dusp6* mRNA was assessed by Real time PCR using specific primers (see “Materials and Methods” section). *Dusp6* expression was normalized to that of *Gapdh* and the data are presented as the means ± SEM from three independent experiments performed on different cultures. ***p < 0.001; **p < 0.01; *p < 0.05.

**Figure 5:**
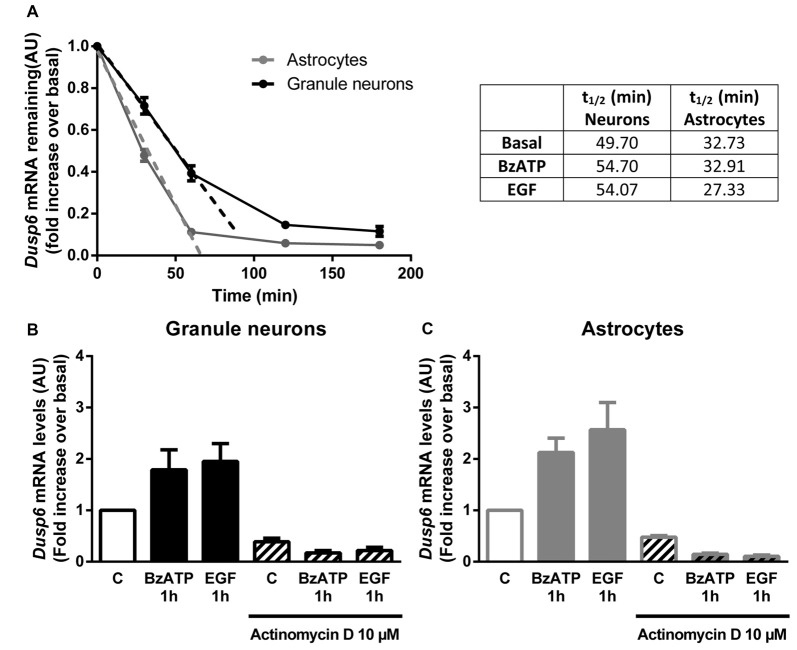
DUSP6 expression is regulated at the transcriptional level.** (A)** Cells were treated with 10 μM Actinomycin D for the indicated times and DUSP6 mRNA levels were measured by RT-Q-PCR and normalized to *Gapdh* mRNA levels. *Dusp6* mRNA half-life (t_1/2_) was calculated by linear regression **(A)**. In other set of experiments cells were stimulated with the effectors, BzATP (300 μM) or EGF (100 ng/mL), and then were treated with Actinomycin D for the indicated times in the continuous presence of effectors. *Dusp6* messenger levels and half-life were estimated as previously described. Half-life (t_1/2_) values are summarized in the upper right table. **(B,C)** Cells were pretreated with 10 μM Actinomycin D for 30 min (astrocytes) or 60 min (granule neurons) previous to be stimulated for 1 h with BzATP (300 μM) or EGF (100 ng/mL) and *Dusp6* mRNA levels were quantified as mentioned previously. Data are presented as the means ± SEM from three independent experiments performed on different cultures.

**Figure 6:**
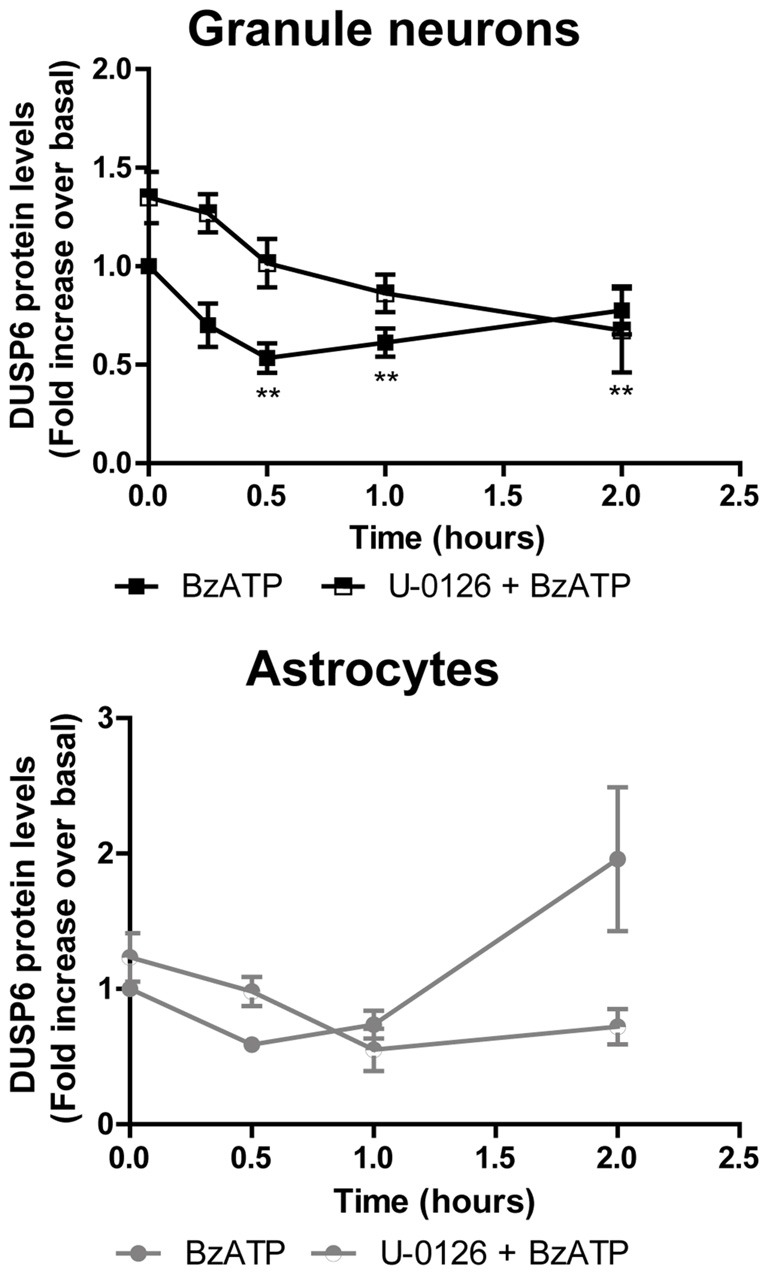
Effect of mitogen extracellular activated kinases (MEK) inhibition on the changes in DUSP6 protein induced by BzATP in granule neurons and astrocytes. Cells were preincubated with or without U0126 (10 μM) for 30 min prior to stimulation with the nucleotide for the times indicated. The blots shown are representative of five independent experiments and the diagrams represent the quantification of the time courses obtained by normalization to the corresponding GAPDH levels. The data are presented as the means ± SEM of five independent experiments performed on different cultures. **p < 0.01.

**Figure 7:**
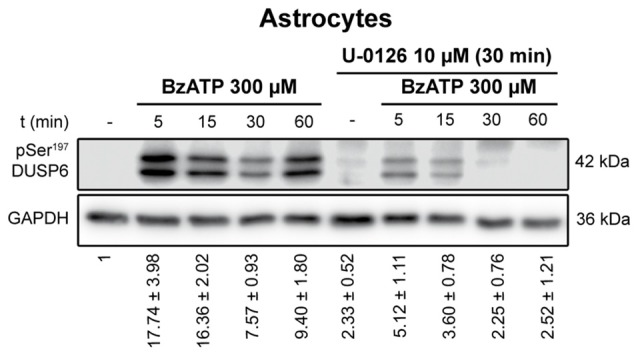
Ser^197^ Phosphorylation of DUSP6 induced by BzATP is dependent on ERK1/2 activation in astrocytes. Astrocytes were preincubated in the presence or absence of U0126 (10 μM) prior to stimulation with BzATP (300 μM) for the times indicated, and the presence of phospho-Ser^197^ DUSP6 was detected in immunoblots. Bands of a representative experiment are shown and the diagrams represent the quantification of the time courses obtained by normalizing to the corresponding GAPDH levels. The data are presented as the means ± SEM from the five independent experiments performed on different cultures.

**Figure 8:**
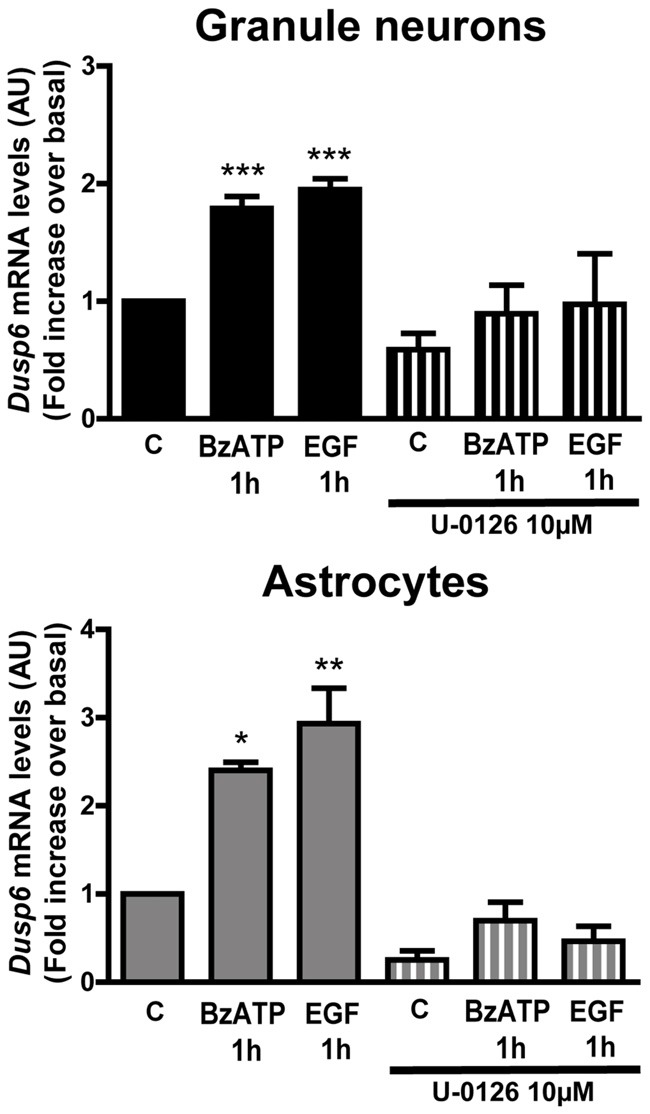
Induction of *Dusp6* expression by BzATP and EGF in granule neurons and astrocytes is dependent on ERK activation. Cells were preincubated with or without U0126 (10 μM) prior to stimulating with BzATP (300 μM) or EGF (100 ng/mL) for 1 h and the expression of *Dusp6* was detected by Real time-PCR (see Figure 3). The *Dusp6* expression was normalized to that of *Gapdh* and the data are presented as the means ± SEM from three independent experiments performed on different cultures. ***p < 0.001; **p < 0.01; *p < 0.05.

**Figure 9:**
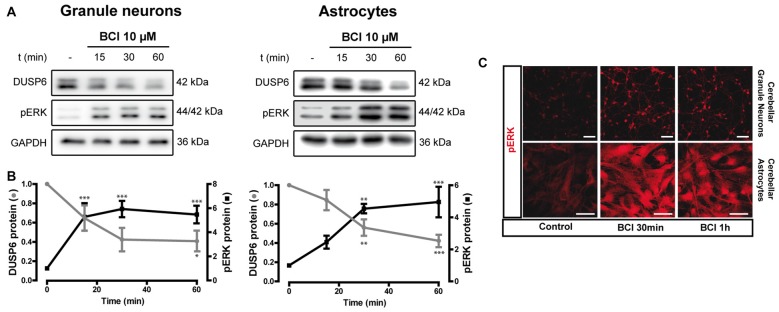
Effect of (E)-2-benzylidene-3-(cyclohexylamino)-2,3-dihydro-1H-inden-1-one (BCI) on the basal levels of DUSP6 and phospho-ERK1/2 in cerebellar granule neurons and astrocytes. **(A,B)** Cells were incubated for different times with BCI (10 μM), a selective DUSP6 inhibitor, and DUSP6 and phospho-ERK were determined in immunoblots. The blots are representative of four independent experiments. Diagrams represent the quantification of the time courses obtained by normalizing to the corresponding GAPDH levels and the data are presented as the means ± SEM from four independent experiments performed on different cultures. **(C)** Cells plated on coverslips were treated with or without BCI (10 μM, 30 min), fixed and the presence of phospho-ERK was detected by immunocytochemistry (see “Materials and Methods” section). Representative images of granule neurons and astrocytes stained for phospho-ERK. Scale bars represent 50 μm. ***p < 0.001; **p < 0.01; *p < 0.05.

**Figure 10:**
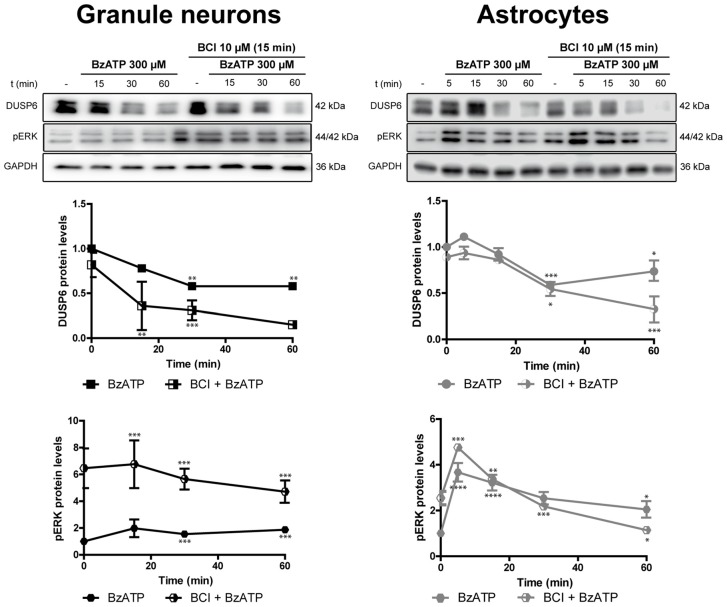
Effect of BCI on the BzATP-induced changes in DUSP6 and phospho-ERK in cerebellar granule neurons and astrocytes. Cells were pretreated with or without BCI (10 μM) for 15 min prior to stimulation with BzATP (300 μM) for the times indicated, and the DUSP6 and phospho-ERK1/2 proteins were determined in immunoblots. The bands are representative of four independent experiments and the diagrams represent the quantification of the time courses obtained after normalizing to the corresponding GAPDH levels. The data are presented as the means ± SEM from four independent experiments performed on different cultures. ****p < 0.0001; ***p < 0.001; **p < 0.01; *p < 0.05.

To determine whether the initial decrease of DUSP6 levels was due to its proteasomal degradation, we assessed the effect of the proteasome inhibitor, MG-132. In cells preincubated with 10 μM MG-132 for 30 min prior to stimulation with BzATP or EGF, there was no initial decay in DUSP6 after short stimulations. Hence, stimulation with these agonists provoked DUSP6 proteasomal degradation. Indeed, preincubation of cells with the proteasome inhibitor enhanced the basal levels of the protein phosphatase (Figure 3), an effect that was more pronounced in astrocytes in which a 2-fold increase over basal levels was observed compared to the 30% increase in granule neurons. Immunofluorescence images in Figure 3C show that DUSP6 staining is mainly cytosolic, spreading out to the cell body and processes.

In order to examine the mechanism responsible for the recovery of DUSP6 protein levels after prolonged BzATP or EGF stimulation (second phase), we performed Q-PCR to study *Dusp6* mRNA expression. Stimulation of cerebellar granule neurons or astrocytes with BzATP and EGF caused a rapid increase in *Dusp6* mRNA expression (at 30 min), which reached a maximum 2–3 fold increase after a 1 h stimulation (Figure 4). However, while longer stimulation with BzATP (4–6 h) returned the *Dusp6* mRNA to basal levels, a similar incubation with EGF maintained high levels of mRNA transcripts in glial cells. Involvement of transcriptional mechanisms in BzATP and EGF effects was examined by using Actinomycin D, a transcription inhibitor (Figure 5). Actinomycin D (10 μM) gradually decreased the basal expression of *Dusp6* mRNA in treatments up to 60 min in both cellular types. Although the RNA Half-life was different, being 49.70 and 32.91 min in granule neurons and astrocytes, respectively (Figure 5A). The half-life of messengers was not modified by any effector neither in neurons nor astrocytes. Pretreatment with Actinomycin D completely blocked the increase in *Dusp6* mRNA levels mediated by BzATP and EGF (Figures 5B,C).

### ERK Proteins Regulate the Turnover and Expression of DUSP6

To determine whether activation of ERK pathway is required for DUSP6 degradation, we tested the effect of the mitogen extracellular activated kinases (MEK) inhibitor, the compound U0126. Treating cells for 30 min with 10 μM U0126 prevented the decrease of DUSP6 observed after a 30 min stimulation with BzATP in both cell types (Figure 6). Noticeably, U0126 treatment did not fully prevent DUSP6 protein decline in granule neurons, although it extensively reduced the percentage decrease from 50% to 30% after 30 min of BzATP stimulation (Figure 6). This was indicative of additional mechanisms taking place together with ERK signaling to target DUSP6 for degradation. Moreover, the recovery of the phosphatase levels normally seen 1–2 h after stimulation with the nucleotide was also impeded by this inhibitor, indicating that ERK activation was required for protein turnover. Similar results were obtained for the growth factor in both neurons and glial cells (data not shown). To assess whether ERK directly phosphorylates DUSP6, thereby targeting the phosphatase for proteasome degradation, we used an antibody against pSer^197^ DUSP6. BzATP stimulation induced a strong increase in Ser^197^ phosphorylation of DUSP6 in astrocytes, which was considerably lower (86%) in cells pretreated with the MEK1 inhibitor, U0126 (Figure 7). The sensitivity to the MEK inhibitor indicated that ERK1/2 could be responsible for the Ser^197^ phosphorylation of DUSP6. However, no Ser^197^ phosphorylated DUSP6 was detected in granule neurons.

The results obtained with MEK inhibitor, U0126, also indicated that ERK activation might induce *Dusp6* gene expression. To investigate the participation of ERK in this process, cells pretreated with 10 μM U0126 were stimulated with BzATP or EGF, and in these cells there was no increase in the mRNA encoding for DUSP6 in cells exposed to U0126 1 h after stimulation with BzATP or EGF (Figure 8). Hence, ERK activation was apparently required to induce *Dusp6* gene expression, the subsequent translation of these mRNA transcript and the synthesis of new DUSP6 protein.

### Effect of DUSP Inhibitor on ERK Phosphorylation in Basal and BzATP Stimulated Cells

There is evidence that DUSP6 could be one of the protein phosphatases responsible for ERK1/2 dephosphorylation, contributing to negative feedback regulation. To test whether DUSP6 regulates ERK activation and *vice versa*, we assessed the effect of a selective allosteric inhibitor of DUSP6, BCI (NCS 150117; Molina et al., 2009). Treatment of astrocytes or neurons with 10 μM BCI enhanced ERK phosphorylation over time in a manner that was inversely correlated to the decay in basal DUSP6 levels (Figures 9A,B). In the case of glial cells, the changes were more gradual than those observed in neurons. Indeed, maximal ERK activation in neurons was observed after 15 min in the presence of 10 μM BCI, whereas in the case of glial cells maximal ERK phosphorylation was delayed around 30–60 min of incubation with the inhibitor. The phosphorylation of ERK induced by BCI was also corroborated by immunocytochemistry (Figure 9C) revealing a predominant cytosolic localization. The treatment with DUSP inhibitor also affected the responses elicited by the nucleotide (Figure 10). In granule neurons, pretreatment with BCI (10 μM, 15 min) potentiated the decrease in levels of DUSP6, agreeing with the predominant ERK phosphorylation that accounted for 6 to 7-fold increases and prevailed over the 2-fold increase obtained with BzATP alone (Figure 2). In contrast, pretreatment with DUSP inhibitor in astrocytes did not affect ERK activation kinetic displayed by the nucleotide.

## Discussion

**Figure 11:**
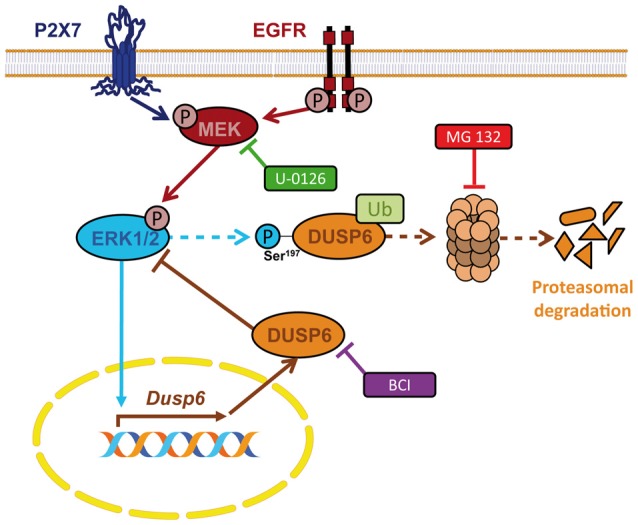
Schematic representation for the regulation of DUSP6 by P2X7 and EGF receptors in neurons and astrocytes. P2X7 and EGF receptors promote biphasic regulation of the expression of the ERK-selective phosphatase DUSP6. Short-term stimulation triggers DUSP6 degradation via proteasome. ERK1/2 proteins could be involved in the targeting of the phosphatase to be sent to the proteasome. Long-term stimulation induces *Dusp6* gene expression and requires ERK1/2 signaling.

In the present study we demonstrate the presence of the dual specificity protein phosphatase DUSP6 in granule neurons and astrocytes from the rat cerebellum, and its regulation by nucleotide P2X7 and EGF. Stimulation with a P2X7 specific nucleotide and of EGF receptors regulates the levels of DUSP6 according to biphasic kinetics. This regulation involves an early degradation phase that takes place following a short stimulation, and that represents a feedforward mechanism to amplify and prolong ERK1/2 signaling. This is followed by a second phase in which there is a recovery in DUSP6 protein levels that works as a negative feedback mechanism to restore basal ERK1/2 activity. Interestingly, ERK1/2 proteins are involved in the biphasic modulation of DUSP6 as ERK activity is required for both degradation and for the recovery phase. The data reported here indicate that this biphasic regulation seems to work as a general and universal mechanism shared by different types of extracellular signals, and that it operates similarly in both native primary cells and established cell lines (Jurek et al., 2009; Ndong et al., 2014; Ham et al., 2015). Thus, we conclude that both P2X7 and EGF receptors are key elements that shape the final biological outcome of ERK1/2 signaling through the regulation of the ERK-selective phosphatase DUSP6 in neurons and glial cells (Figure 11).

In both cell models, granule neurons and astrocytes, the DUSP6 protein undergoes rapid degradation after short stimulations with the P2X7 agonist or EGF. The half-life of DUSP6 degradation after short stimulations of 15–30 min is similar in both cell types, in accordance with previous data (Jurek et al., 2009). The proteasome inhibitor MG-132 prevents DUSP6 degradation, indicating the ubiquitin-proteasomal pathway is involved in this process. The degradation of DUSP6 protein is inversely correlated with the phosphorylation of ERK1/2 and indeed, as the DUSP6 inhibitor BCI enhances ERK1/2 phosphorylation, this protein phosphatase appears to be involved in regulating basal cytosolic ERK activation (Donaubauer et al., 2016). The specificity of BCI as an inhibitor of DUSP6 was first reported by studying the FGF-mediated increase in ERK signaling in zebrafish embryos (Molina et al., 2009). Yet BCI is also known to inhibit the nuclear and inducible phosphatase DUSP1, which exhibits broader substrate selectivity and interacts with ERK, p38 and c-Jun N-terminal kinases (JNK). However, given that enhanced ERK phosphorylation was mainly observed in the cytoplasmic compartment, we conclude that BCI is likely to have inhibited DUSP6 in both granule neurons and astrocytes.

Another interesting aspect of DUSP6 degradation in response to BzATP and EGF stimulation is the need for ERK activity, with DUSP6 degradation attenuated in the presence of the inhibitor of MEK1, U0126. Hence, ERK1/2 proteins participate in a self-regulatory loop acting via direct targeting of DUSP6, probably through the DUSP6 phosphorylation on Ser^197^, that was dampened by the MEK inhibitor. However, U0126 only partially prevented the loss of the DUSP6 protein, as noted in BzATP stimulated granule neurons. Moreover, no DUSP6 phosphorylation was evident in these neurons after BzATP stimulation. Therefore, we cannot exclude the influence of other kinases and intracellular pathways in the regulation of DUSP6, or even the involvement of other residues susceptible to phosphorylation. Additional work is necessary to further confirm that DUSP6 phosphorylated in Ser^197^ is subsequently ubiquitinated in astrocytes. In this regard, DUSP6 undergoes similar regulation following growth factor stimulation in systems overexpressing Ras mitogenic signaling and systems with inducible DUSP6 expression (Zeliadt et al., 2008). Accordingly, Ser^159^ and Ser^197^ residues in the N-terminal domain of DUSP6 were first recognized as targets for ERK1/2 and mTOR-mediated phosphorylation, promoting DUSP6 degradation in response to serum in fibroblasts ectopically expressing DUSP6 (Marchetti et al., 2005; Bermudez et al., 2008). In endothelial cells overexpressing PDGF receptors, DUSP6 underwent rapid degradation similar to that observed here in neurons and astrocytes when stimulated with BzATP or EGF. The Ser^174^ residue was identified as a target for ERK1/2 phosphorylation and it was responsible for ubiquitin-ligase recruitment (Jurek et al., 2009), similar to the regulation of other DUSP family members. ERK1/2-mediated phosphorylation of the DUSP1 inducible phosphatase at different residues may promote either degradation or stabilization (Brondello et al., 1999; Lin and Yang, 2006). In addition, signaling kinases other than ERK1/2 can regulate DUSP1, such as PKCδ, driving its degradation via the ubiquitin-proteasome pathway in a hippocampal cell line and in cortical neurons during glutamate-induced excitotoxicity (Choi et al., 2006). These data indicate that DUSP proteins are tightly regulated and that they are submitted to multiple levels of post-translational regulation. Indeed, perhaps little is still known about the specific regulatory mechanisms that operate for particular stimuli or in specific cell types.

In terms of DUSP6 recovery, it must be taken into account that although *Dusp6* is not an immediate early gene, its expression is transient and it peaks rapidly after 1–2 h of P2X7 agonist or EGF stimulation, occurring in the same time window to that found for other growth factors (Zeliadt et al., 2008; Jurek et al., 2009). Studies carried out with the transcriptional inhibitor, Actinomycin D, clearly supported that *Dusp6* transcriptional induction appears to be the mechanism responsible for the increases in DUSP6 expression mediated by BzATP and EGF in both cellular models. Indeed, *Dusp6* expression is influenced by the Ets-1 and Ets-2 transcriptional regulators, known targets of ERK1/2-mediated signaling (Ekerot et al., 2008; Jurek et al., 2009). Nevertheless, the recovery phase occurs much more slowly in BzATP stimulated granule neurons, as this agonist prolongs DUSP6 degradation for 1–2 h. Indeed, BzATP stimulation of granule neurons seems to be less effective in supporting DUSP6 synthesis and in promoting the wave of expression that restores basal DUSP6 protein levels after 2–4 h.

The delay in DUSP6 recovery helps maintain ERK signaling active for longer, suggesting that neural P2X7 receptors amplify cytosolic ERK signaling and redirect ERK signaling to their cytosolic targets for longer periods. The physiological meaning of transient vs. sustained ERK1/2 activation may depend on the model under study and the subcellular compartment in which ERK is activated. Prolonged ERK activation favors the survival of macrophages subjected to hypoxia, although in this case the down-regulation of DUSP6 activity was essential for survival, modulating the balance between pro- and anti-apoptotic Bcl-2 proteins (Nyunoya et al., 2005). However, in other models prolonged ERK signaling are correlated to cell death in different models, as occurs in granule neurons submitted to apoptotic stimuli (Subramaniam et al., 2005). Here, DUSP6 inhibition by BCI clearly compromises cell survival of both granule neurons and astrocytes when such inhibition exceeds 1–2 h or when the concentration used exceeds 10 μM. Therefore, DUSP6 expression is crucial to avoid the apoptotic effect of sustained ERK1/2 signaling, as described in astrocytes exposed to glutamate excitotoxicity in the ischemic brain (Szydlowska et al., 2010). Along similar lines, the detrimental effect of elevated ERK signaling in cortical neurons induced by the DNA alkylating agent cisplatin is due to the loss of DUSP6 phosphatase activity (Gozdz et al., 2008). The protective role of DUSP6 in mechanical allodynia has been also reported. Induction of DUSP6 expression by cannabinoid receptors seems to be essential to end elevated ERK signaling during mechanical allodynia in spinal neurons and glial cells (Landry et al., 2012). As well, dysregulation of DUSP6 prevents spontaneous resolution of acute postoperative pain and drives its transition to persistent pain via prolonged neuronal and microglial MAPK activation on spinal cord (Saha et al., 2013).

Regulation of cytosolic ERK activity by DUSP6 is also critical in differentiation. NGF suppresses ERK signaling in PC12 cells by inducing *Dusp6* and this directly drives terminal differentiation into a neuronal phenotype (Camps et al., 1998). Interestingly, constitutive DUSP6 levels are elevated in granule neurons during their differentiation in culture, whereas DUSP6 declines after 1 week of culture, coincident with the onset of P2X7 receptor expression (unpublished results). This is consistent with the P2X7 receptor behaving as a key signal to terminate differentiation. In the presence of P2X7 antagonists, neurite outgrowth is enhanced in hippocampal neurons and neuroblastoma cells (Díaz-Hernandez et al., 2008; Gómez-Villafuertes et al., 2009). Moreover, P2X7 receptor stimulation has a trophic effect on cerebellar astrocytes, which involves PKC/PKD activation and that could be involved in astrogliosis (Carrasquero et al., 2010).

In conclusion, nucleotide P2X7 receptors behave like growth factors in terms of the fine-tuning of ERK1/2 MAPK signaling, acting through the regulation of the DUSP6 protein phosphatase. The biphasic kinetics of DUSP6 regulation involves an early degradation step that prolongs ERK1/2 activation, followed by the synthesis of new DUSP6 protein to terminate ERK1/2 signaling. Both processes occur in an ERK1,2–dependent manner and therefore, ERK1/2 regulates its own activation via DUSP6 activity through feed-forward and negative feed-back mechanisms (Figure 11). This regulation ensures the reduction in ERK phosphorylation necessary to allow the cell to respond to a subsequent stimulation, apparently functioning as a common universal mechanism.

## Author Contributions

MJQ is the recipient of a FPU fellowship and conducted most of the experiments performed in granule neurons. JCG-R is the recipient of a FPI fellowship and conducted most of the experiments performed in astrocytes. VM contributed to the experimental part of the study. FO and MTM-P contributed to the preparation of the figures and discussion of the results. EGD and RP-S contributed to the design and writing of the manuscript. All the authors analyzed the data, revised the manuscript and approved the final manuscript.

## Conflict of Interest Statement

The authors declare that the research was conducted in the absence of any commercial or financial relationships that could be construed as a potential conflict of interest.

## Abbreviations

BzATP: 2′(3′)-O-(4-Benzoylbenzoyl)adenosine 5′-triphosphate
CaMKII: Calmodulin-dependent Protein Kinase II
DMEM: Dulbecco’s modified Eagle’s medium
DUSPs: Dual specificity phosphatases
EGF: Epidermal growth factor
ERK: Extracellular signal regulated kinases
FCS: Fetal calf serum
GPCRs: G protein–coupled receptors
GAPDH: Glyceraldehyde-3-phosphate dehydrogenase
JNKs: c-Jun N-terminal kinases
MAP kinases: Mitogen-activated protein kinases
MEK: Mitogen extracellular activated kinases
MKPs: Mitogen-activated protein kinase phosphatases
PKC: Protein kinase C
RTKs: Receptor tyrosine kinases.
